# The first steps towards a diverse and inclusive EBMT: a position paper

**DOI:** 10.1038/s41409-022-01586-w

**Published:** 2022-02-04

**Authors:** S. Montoto, J. A. Snowden, C. Chabannon, S. Corbacioglu, R. de la Camara, H. Dolstra, R. Greco, A. Gusi, N. Hamad, M. Kenyon, N. Kröger, M. Mohty, J. Murray, A. Mueller, B. Neven, R. Peffault de Latour, Z. Peric, I. Sánchez-Ortega, A. Sureda, B. Verhoeven, A. Villar, I. Yakoub-Agha

**Affiliations:** 1 Department of Haemato-oncology, St. Bartholomew’s Hospital, Barts Health NHS Trust, London, UK; 2 Department of Haematology, Sheffield Teaching Hospitals NHS Foundation Trust, Sheffield, UK; 3 Department of Oncology & Metabolism, Medical School, The University of Sheffield, Sheffield, UK; 4 Centre de Thérapie Cellulaire & Inserm CBT-1409, Institut Paoli-Calmettes, Centre de Lutte Contre le Cancer, Marseille, France; 5 Department of Pediatric Hematology, Oncology and Stem Cell Transplantation, University of Regensburg, Regensburg, Germany; 6 Hospital de la Princesa, Madrid, Spain; 7 Department of Laboratory Medicine-Laboratory of Hematology, Radboud University Medical Center, Nijmegen, The Netherlands; 8 Hematology and Bone Marrow Transplantation Unit, IRCCS San Raffaele Scientific Institute, Milan, Italy; 9 EBMT Executive Office, Barcelona, Spain; 10 Department of Haematology, St. Vincent’s Hospital Sydney and St. Vincent’s Clinical School Sydney, University of New South Wales, Sydney, NSW Australia; 11 Department of Haematological Medicine, King’s College Hospital NHS Foundation Trust, London, UK; 12 Department of Stem Cell Transplantation, University Hospital. Hamburg, Hamburg, Germany; 13 Service d’Hématologie Clinique et Thérapie Cellulaire, Sorbonne University, Saint Antoine Hospital and INSERM UMRs 938, Paris, France; 14 Haematology and Transplant Unit, Christie Hospital NHS Foundation Trust, Manchester, UK; 15 Unité d’immuno-hématologie pédiatrique, Hopital Necker, assistance publique hopitaux de Paris, Institut IMAGINE, Université de Paris, Paris, France; 16 BMT Unit, French Reference for Aplastic Anemia and PNH, Hôpital Saint Louis, Paris, France; 17 University Hospital Centre Zagreb, School of Medicine, University of Zagreb, Zagreb, Croatia; 18 Clinical Hematology Department, Institut Català d’Oncologia-Hospitalet, Barcelona, Spain; 19 Patient Advocacy Committee, EBMT Executive Office, Barcelona, Spain; 20 CHU de Lille, Univ Lille, INSERM U1286, Infinite, 59000 Lille, France

**Keywords:** Health care, Health services

## Background and initial progress

Equality, diversity and inclusion (EDI) are now highly topical in culture, society and medicine. There is a recognition that sociocultural norms and current systems have contributed to implicit and explicit forms of discrimination based on several characteristics including but not limited to gender, gender identity, sexual orientation, age, disability, nationality, ethnicity, culture, religion, marital and family status, as well as education and professional background. Inclusion is a term that has come to mean celebration of those previously considered as ‘other’, recognising their lived experiences and perspective as an asset that benefits the individual, organisation and society at large.

Aspirations for EDI are central to the Mission and Vision of the European Society for Blood and Marrow Transplantation (EBMT) (https://www.ebmt.org/ebmt/our-mission-vision-values) as well as being core values of staff, members and patients. However, diversity amongst a society membership does not necessarily mean such an organisation is diverse and inclusive. Questions are reasonably asked if, for example, the leadership of an organisation at various levels does not reflect the diversity of its membership, or if invited speakers at educational meetings do not reflect the diversity of the attendees.

EDI has been an increasingly important topic for discussion in the EBMT Executive Committee (ExCom), Board of Association (BoA) and Scientific Council (SC) meetings since 2018, when the recognition of gender imbalance was followed by decisions to take proactive steps as an organisation to identify the contributing factors in order to redress them. In 2019, the EDI Working Group was created, comprising volunteers from the membership, ExCom, BoA, SC, Patient Advocacy Committee and staff to form a gender-balanced and diverse committee. The group incorporated guidance from external consultancy which was sought to evaluate and advise on EDI dimensions. In 2020, initial quantitative and qualitative surveys were sent to the membership and staff. In addition, the annual EBMT meeting began including special sessions focussed on EDI and health disparities. The results of these initial surveys and associated developments are summarised in the EDI section of the EBMT website https://www.ebmt.org/equality-diversity-inclusion.

As with many other national and international scientific organisations, it is in the best interests of the EBMT as a society to proactively champion EDI and lead by example by reaching out and maximising the potential of all members of the organisation. This position paper summarises the progress made, further steps that are necessary, and gradual changes in culture for the EBMT to ensure its success in becoming truly inclusive and diverse.

## Data monitoring and surveys

**Fig. 1:**
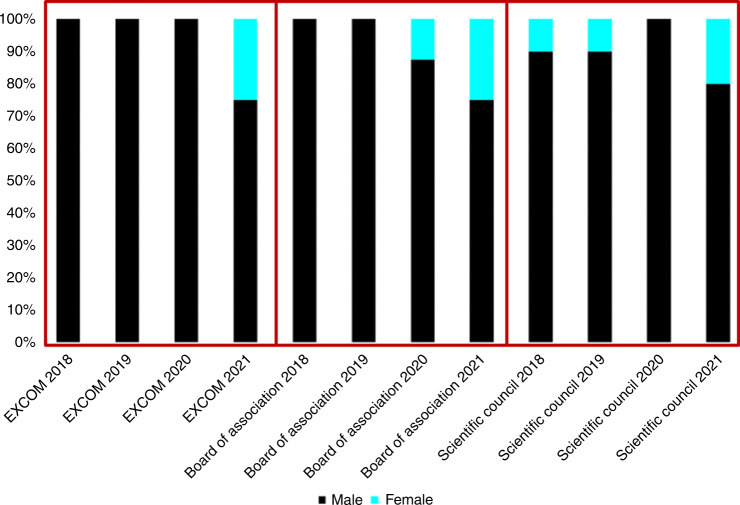
EBMT leaderdhip gender balance. Distribution of assumed gender balance in the EBMT Excom, Board of Association, and Scientific council during the 2018–2021 period.

**Fig. 2:**
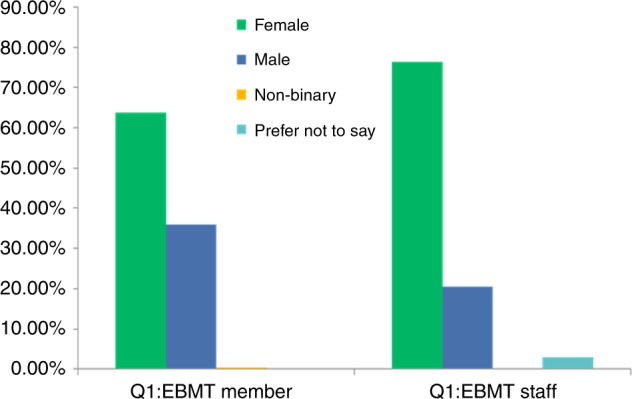
Gender distribution of EBMT members and staff. Distribution of self-assigned gender amongst EBMT members and staff.

Diversity considers gender, sexual orientation, ethnicity, religion, disability, pregnancy and parental rights. In many countries, there are legal frameworks against discrimination in relation to ‘protected characteristics’. Gender distribution is thus not the only measure of diversity, but gender imbalance is the most easily demonstrable example of inequity. There has been clear evidence of this in the EBMT leadership over the last 4 years (Fig. 1). The EBMT BoA and SC are composed of the ExCom (President, Secretary, Treasurer and elected President, in the election years) and the 10 Chairs of EBMT Working Parties (WP) (https://www.ebmt.org/ebmt/who-we-are). These positions are elected by the EBMT membership: each centre can cast one vote but only the principal investigator (PI) of each EBMT centre has voting rights. Between 2018 and 2021, amongst 21 candidates for ExCom members and WP Chair elections, only 5 (24%) were women. In 2018, there were no women in the ExCom or BoA and only one woman (i.e., two successive WP Chair terms) amongst 10 EBMT SC members. With proactive strategies, such as the constitution of its former EDI Working Group as a formal Committee of the organisation, the establishment of an internal (staff) taskforce to lead on EDI implementation and the review of by-laws to update them in line with good practice, some progress has been made. The 2021 elections have partially addressed this gender imbalance with women representing 25% of the ExCom, BoA and SC membership, including a woman in the position of president-elect. However, there is still a significant disparity when compared to the composition of EBMT staff, which is selected according to employment law, and of the broader EBMT membership, which demonstrates gender parity reflecting the broader population (Fig. 2). Of note, the data on gender distribution amongst ExCom members and WP Chairs is based on assumed gender, rather than self-identified gender as, unfortunately, ExCom members and WP Chairs have not been so far asked about their gender identity. Similarly, no information regarding self-identified gender distribution amongst EBMT centre PIs is available. This is relevant, as ExCom members and WP Chairs are ultimately elected by the centre PIs. The need to collect and monitor this data is now clearly acknowledged in the EBMT and this information should be available in the near future.

**Fig. 3:**
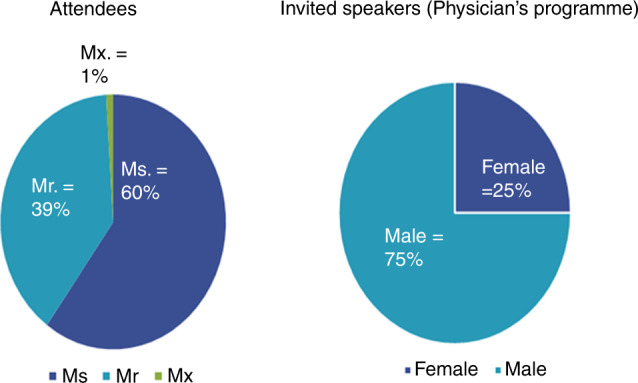
Gender distribution of attendees and invited speakers at EBMT Annual Meeting 2020. Self-identified gender distribution of attendees and assumed gender distribution of invited speakers at the physicians’ programme at the EBMT annual meeting of 2020.

Many professional healthcare organisations have recently recognised issues with gender equity as detrimental to their membership and goals. They have also grappled with this issue at their education meetings. This is most reported in the context of gender representation and participation metrics comparing attendees, speakers and acknowledgement of speakers [1]. Data derived from the EBMT annual meetings show that there is a gender imbalance in invited speakers when compared with the gender distribution of attendees (Fig. 3). Other EDI principles including representation of minority groups within the EBMT are yet to be evaluated. In a multinational organisation like the EBMT, this issue is more complex and requires consideration of inclusive strategies that do not solely rely on proportional representation.

## Discussion: challenges and future steps

**Fig. 4:**
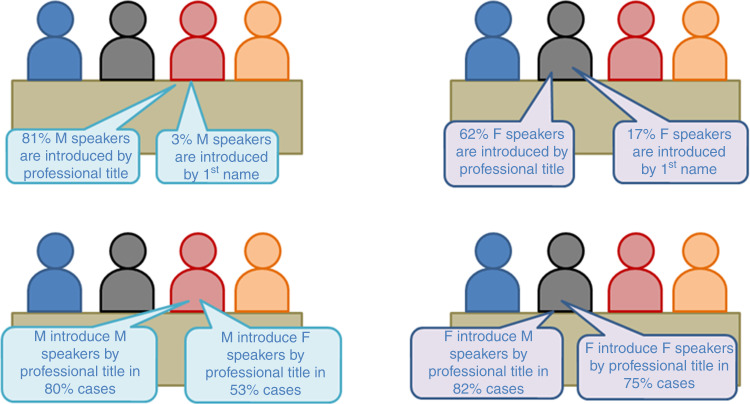
Introduction of speakers at the 2017 and 2018 annual American Society of Clinical Oncology (ASCO) meetings according to speaker and moderator gender. Adapted from Duma et al. J Clin Oncol. 2019. F female, M male.

The fact that some population groups are, either consciously or unconsciously, discriminated against is not a novel concept in healthcare. The discourse around this topic has matured with an intersectional lens. Gender intersects with other factors that drive inequity, discrimination and marginalisation, such as ethnicity, socioeconomic status, disability, age, geographic location and sexual orientation. These are compounded by each other for those represented in multiple disadvantaged groups, for example, women of colour. Sociocultural stereotypes and ingrained systemic structural biases perpetuate an imbalance in representation, academic progression and power to affect change. This lack of diversity in representation and consequently thought has an impact on the broader community. Unconscious bias against under-represented groups can be manifested by subtle micro-aggressions such as belittling, undermining, ignoring or dismissing the views and contributions of other members of the team, as well as the not so subtle aggressions such as sexual harassment [2–5]. An example of such subtle discrimination is illustrated by the way female and male speakers are introduced at international meetings (Fig. 4). The implementation of strategies to redress these injustices is the next phase of evolution in the culture of medicine. The effect of gender, ethnicity and non-native accents on how both students and faculty are assessed has been reported [6, 7]. Career progression and academic recognition are more challenging for women, people from ethnic minorities or other under-represented groups. The imbalance in gender distribution of invited speakers to national and international meetings is well reported in other societies [8]. Discounting certain groups as leaders (by conscious or unconscious bias, or just familiarity) significantly reduces the pool of potential committee candidates, increasing the risk of selecting a candidate solely due to the lack of alternative contenders. It is crucial to realise that a fair representation of women or minority/under-represented groups in governance bodies is not a solution to discrimination or bias without an inclusive approach where there is genuine recognition, appreciation and respect for their contributions.

These issues are by no means unique to the EBMT, but the society has resolved to lead in creating meaningful and enduring change. Other societies have led the way in looking inward and recognising EDI issues [9] and are actively working to address them. The American Society of Hematology implemented a Minority Recruitment Initiative in 2003 (https://www.hematology.org/awards/minority-recruitment). The British Society of Haematology reported a roughly even gender split amongst speakers at the 59th Annual Scientific Meeting in 2019, with almost one in five of the speakers of black, Asian and minority ethnic origin. In 2020, the Royal College of Physicians in the UK commissioned an external review on EDI, one of the objectives of which was to make recommendations to the Board of Trustees and Council on the necessary changes (https://www.rcplondon.ac.uk/projects/rcp-equality-and-diversity-review).

The EBMT has similarly resolved to enact positive change and has implemented some strategies. The former EDI Working Group has become a formal Committee of the organisation. The EBMT has also established an internal (staff) taskforce chaired by the Chief Executive to lead on EDI implementation and report to the Committee and has embarked on a review of all recruitment and employment processes. An EDI policy is in progress, the EBMT code of conduct is being updated and the EBMT has begun a review of by-laws to update them in line with good practice. In addition, EDI-related sessions at the annual meeting will continue. These were originally introduced in the 2020 and 2021 programmes and underscore the commitment of the EBMT to address EDI issues visibly, proactively and effectively, with a realistic view to achieving gradual but permanent change.

At its October 2020 meeting, the EBMT Board adopted a mandate to aim for at least 25% female representation among its Officers and WP within 3 years (i.e., by the end of 2023). This ‘ambition’, albeit arguably limited, was actually achieved in the elections of 2021. Despite this, there remains a need to empower and support women and minorities into more senior roles, with a goal of increasing diversity and balance to reflect effective representation across ethnicities, sexualities, genders and abilities at all levels within the EBMT.

The task ahead for EDI in the EBMT is not an easy one. It takes time to change attitudes and behaviours across a wide geographical membership drawn from a large number of countries with significant social, cultural, political and religious heterogeneity. The governance bodies of the EBMT acknowledge and respect these differences and are determined to take a firm standpoint and commit to implementing real change, rather than just well-meaning statements and cosmetic measures. New technologies, with virtual/remote ways of working (widely used since the COVID pandemic), will likely help to promote EDI, by facilitating participation by those with a disability, carer commitments or other barriers including geographic distance and economic disadvantage. The EBMT is holding its 2022 annual meeting in a ‘hybrid’ format, which may help to inform further plans. Ultimately, the efficacy of any efforts to improve EDI in the EBMT so that it becomes truly diverse and inclusive are best informed by evidence through data monitoring, audit and regular review of all aspects of EDI.

In summary, supported by monitoring data, gender inequality has been accepted as an issue within EBMT, and, over the last 4 years, some progress has been made since it developed a focussed approach. However, gender is only one aspect of the broader spectrum of EDI within the EBMT where there is limited information and further work is warranted in evaluating and monitoring progress across all aspects of EDI within our professional organisation. Working towards a more inclusive EBMT will also support a better understanding of health inequalities in the diverse patient communities we serve.
